# How much does culture matter for preferences for redistribution? Evidence from panel data on immigrants in Germany

**DOI:** 10.1371/journal.pone.0327372

**Published:** 2025-07-30

**Authors:** Olga Griaznova

**Affiliations:** Department of Political and Social Studies, University of Pavia Corso Strada Nuova, Pavia, Italy; University of Naples Federico II: Universita degli Studi di Napoli Federico II, ITALY

## Abstract

This study addresses a long-debated question in the humanities: does culture matter? This issue gains prominence in times of intense migration, when disputes about migrants integration and their impact on the political processes become central in political discourse. Using data from the German socio-economic panel (GSOP), the study brings together empirical evidence about preferences for redistribution of both the German population and migrants originated from different welfare regimes. Focusing on the political integration of migrants, this article explores whether immigrants from Poland, Turkey, Kazakhstan and Russia adopt preferences for redistribution of native Germans during their life in Germany. By applying OLS and fixed effects models, the study tests theoretical scenarios of the adaptation of migrants in the host country and draws conclusions about the complexity of the integration process.

## Introduction

“Is it possible that living under a specific system leads to adaptation of preferences?”. This is the key question that Alesina and Fuchs-Schündeln address in their paper “Good-Bye Lenin (or Not?): The Effect of Communism on People’s Preferences” [1, p. 1507]. This influential study exploited the exogenous shock induced by the reunification of Germany to investigate the causal effect of living in a given economic and political regime on preferences for redistribution. While Alesina and Fuchs-Schündeln discussed the long-lasting effect of Communism, this research focuses on the long-lasting effect of the culture of origin on immigrants’ preferences in Germany. I ask the following question: “Do immigrants adapt their redistribution preferences to the new institutional context of the host country or do they hold on to the attitudes shaped in their country of origin?”. The question about adaptation of preferences for redistribution is of particular interest nowadays because of their political and electoral importance. And this question seems to be even more important if we take into account increasing migration flows in Europe and the inclusion of migrants in the electoral process. The aim of this study is to build on previous studies to extend an explanatory framework in a new context, to offer a test of the reliability of existing explanatory models and to make a step ahead to understand patterns of immigrants’ adaptation in the host country.

Previous findings indicate that the culture of country of origin shapes migrants’ preferences for redistribution: the stronger the demand for redistribution in the country of birth, the more likely the immigrant is to favor redistribution [2,3]. Alesiana and Fuchs-Schündeln expanded on this by examining the dynamics of the process, providing evidence that cultural influence persists long after changes in institutional and economic environment. However, they also demonstrated that the culture of the country of residence can modify these preferences, aligning them more closely with the dominant cultural context.

This study builds on previous findings and focuses on the influence of culture over time but in a different context. To be more specific, the study tracks immigrants in Germany over time and estimates the impact of both the culture of origin and destination on their individual preferences. Thus, the study aims to shed light on the mechanism of cultural integration of immigrants in the host country. Using data from the German Socio-Economic Panel (G-SOEP), the study identifies the dynamics of cultural integration under a condition of self-selection among different migrant groups. The results show that labor migrants, particularly from Poland, exhibit a rapid alignment of their preferences with those of native West Germans, driven in part by self-selection. In contrast, migrants from Russia and Kazakhstan display slower adaptation, with partial integration observed in some policy areas, but also a tendency toward separation in others. These findings suggest that the process of adaptation is multifaceted, shaped by both individual migration motivations and the context in the host country. Additionally, the study highlights that changes in the host country’s welfare preferences, such as increased support for social security, can influence both migrant and native preferences, further complicating the understanding of cultural adaptation processes.

## Theoretical framework

### Acculturation theory

Building on the findings about the influence of culture, theories explaining the adaptation of migrants in a new country are especially relevant to this research. J. Berry’s acculturation theory provides a framework for understanding how immigrants adapt and integrate into a new culture. According to Berry [4,5] and Kwak & Berry [6] acculturation theory identifies four possible scenarios for immigrant adaptation, each reflecting the interaction between the culture of origin and the host culture. When immigrants move to a new country, their attachment to either their culture of origin or host culture shape their experience. These different adaptive strategies include: assimilation (abandoning the culture of origin and adopting the host culture), integration (maintaining the culture of origin while adopting the host culture), separation (retaining the culture of origin while rejecting the host culture) or marginalization (abandoning both cultures). While assimilation involves complete adaptation to the host culture at the expense of the culture of origin, integration suggests a more balanced, two-way process, where both cultures influence each other. This distinction is particularly important in our study, as we examine integration rather than assimilation. While both processes may overlap in certain cases, particularly in terms of converging toward host country preferences, our focus is on the adaptive strategy that allows for a retention of the original culture alongside the adoption of some aspects of the host culture.

Several studies have revealed complexities of migrant adaptation to a new cultural environment. Inglehart and Norris argued that “[m]igrants do not wholly reject their cultural roots, it seems, but neither do they fully adopt the values of their host societies” [7, p. 241]. This view is supported by Moreno [8] who compared values of Mexicans who lived in Mexico and in the US with Anglo-Americans living in the US, and by Nicolás [9] who studied migrants in Spain and compared their social and political values with average values in their countries of origin and average values of native Spaniards. Both studies found that migrants’ preferences and values typically fall somewhere between the averages of their home and host countries, reflecting the integration process.

### Self-selection issues

Migrants represent a diverse social group, shaped by unique social, economic, and historical factors that distinguish them from native populations in both their countries of origin and destination. The motivations for migration, as well as the circumstances surrounding relocation, vary significantly across different migrant groups. This distinction has become especially evident in recent years, when, on the one hand, globalization has stimulated intense geographic mobility of highly skilled workers, and on the other, wars and natural disasters have forced people flee their homes. Labor migrants are often self-selected risk-takers who choose to move voluntarily in search of better economic opportunities. These individuals typically have lower preferences for redistribution compared to the average in their countries of origin, as they tend to be self-reliant and less dependent on state welfare. Studies have shown that labor migrants, driven by the desire for upward mobility, are less likely to rely on social benefits [10]. They are often motivated by the potential for economic success and the opportunity to integrate into a new society based on their skills and personal traits [11].

The findings discussed above describe migrants who relocated to another country voluntarily. In contrast, refugees and forced migrants tend to come from more vulnerable socio-economic backgrounds. Their migration is often not motivated by opportunity but by necessity, fleeing war, political instability, or natural disasters. As a result, they are more likely to be dependent on state welfare provision in the host country, shaping their preferences for redistribution in a way that reflects their immediate need for social support rather than cultural attitudes toward redistribution. Since this study focuses on effect of culture and changes in preferences for redistribution, it is essential to single out migration groups that are less biased  in terms of self-selection. Immigrants from Russia and Kazakhstan in 1990^th^, who are mainly native German resettlers, I consider as a least biased case in terms of preferences for redistribution that allows to study integration process and effect of culture best possible way. Ethnic German resettlers from Russia and Kazakhstan, occupies a unique position. These individuals were not risk-takers seeking economic opportunities, nor were they forced migrants. Instead, they were granted the right to resettle in Germany through state-supported channels, where the German government facilitated their migration based on historical ties to the country. Their migration was determined by legal rights and resettlement policies rather than by the dynamics of voluntary migration or forced displacement. As a result, their preferences for redistribution may be more similar to average preferences for redistribution in their host countries.

### Theoretical scenarios of adaptation

Acculturation theory and the self-selection arguments propose the range of possible scenarios of adaptation of immigrants to a new culture. Acculturation theory implies that migrants’ values and attitudes often differ from the average values and attitudes common in the host country, and over time, migrants may experience either assimilation, integration, separation or marginalization. The self-selection argument challenges traditional acculturation assumptions by suggesting that migrants may choose a destination country based on their own values. In this case, the observed assimilation or integration may not be due to the influence of host culture, but rather a result of the migrants’ initial self-selection. Moreover, when studding attitudes towards redistribution, we must take into account that voluntary migrants are often risk-takers, relying on their abilities and being less dependent on social benefits. This factor makes them different from the natives in their country of origin, and their preferences for redistribution may be more liberal compared to those of their home country. In this case, if people with more liberal attitudes migrate to countries with a more liberal welfare regime, their attitudes may align more closely with those of natives, not because of cultural assimilation but due to initial self-selection.

To better understand the adaptation patterns of migrants, it is important to determine how closely migrants’ preferences align with those of natives in the destination country before immigration or at shortly after arrival and follow them over time. To assess the impact of culture, we can track how the attitudes of different migrant groups change over time. This approach guides the research design.

## Method

### Research design

To capture the effect of cultural context, this study traces the adaptation patterns of various migrant groups in Germany and compares them with both West and East Germans. Alesina and Fuchs-Schündeln made an important contribution to the debate on how a changing cultural context affects individual attitudes by studying the adaptation of East Germans to new political and cultural landscape following German reunification. By comparing the attitudes of East Germans with West Germans over time, the authors concluded that West German culture influenced changes in the attitudes of East Germans. However, a closer look at the study, particularly the selection of West Germans as the reference group for comparison, raises the question of whether the convergence in attitudes between East and West German resulted from East Germans adopting West German values, or if it was a mutual, bilateral convergence. This is especially relevant given the unification of the two parts of the country, each with different social and political institutions. Such a context could not only have led to the influence of Western culture on East Germans’ preferences but may have also allowed the culture of East Germany formed under communism to influence West Germans.

To trace the effect of culture more effectively, we need to focus on the adaptation strategies of smaller social groups in a new cultural setting. Migrants, who don’t have significant influence on the dominant culture, are ideal subjects for this study. In addition, to isolate the effect of host country’s culture on individual preferences for redistribution, it is essential to select migrants from countries where welfare regimes and the average demand for redistribution differ from those in the host country.

### Dataset

The German Socioeconomic Panel (G-SOEP) provides an ideal dataset for implementing the research design. It includes data on preferences for redistribution at two time points (1997 and 2002), and covers both natives and immigrants from different countries.

G-SOEP is a comprehensive, representative longitudinal study that began in 1984 in the Federal Republic of Germany and in 1990 in East Germany (the former German Democratic Republic). The dataset contains the data on about 11*,*000 private households and 30,000 respondents. G-SOEP is conducted by the German Institute for Economic Research, DIW Berlin. The survey collects detailed information on all household members, including West and East Germans, foreigners and recent immigrants. Access to the data is granted upon signing a data distribution agreement with DIW, in compliance with the European General Data Protection Regulation (GDPR) and the Federal Data Protection Act (BDSG). Researchers interested in assessing the SOEP data can apply directly to DIW Berlin. The application process involves completing and submitting a data access agreement, which is reviewed and signed by both parties. Once approved, the data are made freely available for research purposes. As a result, special security and data protection measures are in place when using micro-data of the SOEP. Detailed information about SOEP data and the conditions for access can be found on the DIW Berlin website: https://www.diw.de/en/diw_01.c.601584.en/data_access.html. The author confirms that she did not have any special access privileges to the data and that other researchers can access them in the same manner, following the outlined procedures. The data for this study was first accessed in June 2015. The data used in the analysis has been anonymized and is presented in a generalized form in this article.

### Case selection and subsample identification

The research design requires data for individuals who answered questions about preferences for redistribution, participated at least in two waves of the survey, native Germans and immigrants who came to Germany less than 20 years ago. All migrants in this study are permanent residents of West Germany. Individuals who have no information about redistribution preferences, country of origin, sex, age, individual or household income are filtered out. The final sample consists of 8,567 individuals who participated in both survey waves.

There are three key conditions for studying the effect of culture on individual preferences for redistribution. First, there must be a substantial difference in average preferences for redistribution in home and host countries. Only under these conditions it is possible reasonably estimate the effect of time on immigrants’ individual preferences following their relocation. Second, the relocation to the host country must be relatively recent to avoid complete integration of immigrants. Third, subsamples of immigrants must be sufficiently populated in the survey sample to ensure robust analysis.

G-SOEP provides data for four subsamples of immigrants that meet these requirements: immigrants from Russia, Kazakhstan, Poland and Turkey. These data reflect the general structure of migration flows in Germany at the end of the 20^th^ century. Data provided in recent publications [2,3] provide us with evidence that indeed in Poland, Turkey ana Russia average preferences for redistribution are higher than in Germany. Regarding the second identification criteria we can say that immigration was extensive from precisely these countries in the 1990^th^ [12]. So, immigrants from these countries were recent immigrants in 1997. And the survey sample provides substantial number of observations for the study of integration patterns: Turks (915 observations), Pols (279 observations), Russians (147 observations) and Kazakhs (140 observations).

Although immigration from Russia, Kazakhstan, Poland and Turkey occurred simultaneously and was extensive, the nature and causes of these migration flows differ. This difference could generate dissimilar patterns of integration leading to divergent conclusions about the elasticity of preferences for redistribution among migrants. The first factor that distinguishes migrant groups is the reasons for migration. Immigrants from Poland and Turkey are labour immigrants who may be a highly selective group in terms of values and personal traits. Immigrants from Russia and Kazakhstan are mainly ethnic German resettles who had historical reasons for migration and could rely on the support of the German welfare provision upon arrival. The peculiarities of this migration flow suggest an interesting setting for a natural experiment. To justify it better, it is necessary to provide some information about the characteristics of these groups of immigrants.

### Immigration from Former Soviet Union (FSU) countries

Migration from the former Soviet Union to Germany is a distinctive case of ethnic group resettlement. Approximately two million immigrants came to Germany from FSU countries, with the majority coming from Russia and Kazakhstan. This massive resettlement was not spontaneous; it had significant political and historical context. Most of the immigrants were either ethnic Germans (about 90%), people who had German ancestors, or, a smaller share (about 10%) of Jewish quota refugees [13, p. 1440].

Ethnic Germans were settled in the European part of Russian Empire starting from XVI century. Among several waves of migration, the most extensive one was prompted by a decree from Catherine the Great issued in 1764, which granted German immigrants a variety of privileges in Russia [14], [15, p. 6]. The Soviet Census of 1939 indicated a population of 1,427,232 Germans who often lived in distinct communities and maintained different customs compared to Russian population [16, p. 426]. During World War II, ethnic Germans faced suspicion and many were accused of being spies, “concealing enemies” and collaborating with German army [16, pp. 426–427]. In September 1941, the Soviet government condemned German population, leading to several decades of formal restrictions and repression [17, p. 206]. In the fall of 1941, approximately 800,000 Germans were deported from the European regions to Siberia and Kazakhstan, with additional 400,000 displaced during the war. Germans were rehabilitated in 1964 but were not allowed to return to the places where they had previously settled.

Many Germans remained in Siberia and Kazakhstan until the end of the 1980s. Soviet authorities strictly controlled the emigration from the Soviet Union. However, the process of liberalisation of international relations began in 1987, and the emigration reform of 1991 in the Soviet Union together with the German Federal Law Concerning Displaced Persons (“Bundesvertriebenengesetz”, 1953) allowed ethnic Germans to leave the USSR and resettle in Germany. According to the German Federal Law, ethnic Germans were given a special settlement status right upon arrival, which made them eligible for citizenship and provided assistance, including support for labour market integration and access the healthcare and education.

According census data there were 2,038,603 ethnic Germans living in the USSR in 1989, most of them in Russia (842,295 people) and Kazakhstan (957,518 people). During the 1990s, more than 70% of the German population in Kazakhstan and 40% of German population in Russia emigrated. At the time of the Soviet Union collapse, many were driven by economic uncertainty, as well as lingering historical memory of recent repressions. At the same time, according to surveys of ethnic Germans who arrived in Germany between 1990 and 1994, majority of immigrants felt settled and accepted in the countries where they came from [18, p. 49]. In comparison to the Russian context, ethnic Germans in Kazakhstan had an additional reason for emigration: rising Kazakh nationalism and the spread of Islam in the country [14, p. 166]. This situation pushed nearly the entire Germans population in Kazakhstan out of the country for cultural reasons.

The emigration reform of 1991 in the Soviet Union also had an impact on the Jewish population, making it possible for them to emigrate [19]. One year before, East Germany granted asylum to the Jewish population from the Soviet Union who faced discrimination and persecution (Beauftragte der Bundesregierung für Ausländerfragen 1997, 307). They were able to enter Germany using a tourist visa or apply for refugee status. After reunification in 1991, Germany implemented a law to admit refugees to Germany based on quota regulation (Kontingentflüchtlingsgesetz, 1980).

The 1989 Soviet census reported 536,848 Jews in Russia and 18,492 in Kazakhstan. The subsequent census indicated that only 43% of Jews remained in Russia by 2002 and only 674 individuals in Kazakhstan by 1999. Between 1991 and 2000, the German Federal Administration Office confirmed 157,694 applications, of which 128,519 were Jewish immigrants to Germany. An additional 8,535 Jews from the former Soviet Union entered Germany on a tourist visa, remaining in the country without being deported. Unlike ethnic Germans, post-Soviet Jewish emigration was spread across three countries: the United States and Israel were preferred destinations, while Germany hosted around 9% of Jewish emigration from FSU countries [13, p. 1440]. This migration flow in Germany was not as intense as the flow of ethnic Germans, but it cannot be ignored. In addition, the legal side and the motive of this migration were generally no different from the migration of ethnic Germans. Key motivations for emigration included antisemitism, ethnic tension, political instability, economic crisis, and ecological catastrophes [19, p. 36].

Both ethnic Germans and Jewish migrants from the FSU faced similar historical experiences, shared common ancestry, and belonged to a discriminated minority groups. They originated from the same regions and spoke Russian as their primary language. Legal admission criteria and integration support in Germany made these groups comparable in many respects, distinguishing them from labour immigrants and asylum seekers [18, p. 51]. For this research, the most critical aspect is the similarity in motivation for emigration shared by both ethnic Germans and Jews, which stem from historical experience and legal conditions. These factors minimise potential biases resulting from both by self-selection into migration and the choice of host country.

The preferences for redistribution among immigrants from Russia and Kazakhstan can be considered as a non-biased case, as this migration flow consists predominantly of ethnic Germans and Jewish quota refugees. The historical reasons for emigration of ethnic Germans and Jews from these countries, along with the institutional support they received in Germany upon arrival, suggest that their decision to emigrate was not influenced by individual traits or by similarity between the country of origin and the destination. This case also allows for the avoidance of a double selection bias, which is typically driven by labour market aspirations and migrant networks that encourage migration [13, p. 1452].

Comparing the preferences of immigrants from Kazakhstan and Russia with those of West and East Germans provides unbiased estimates of preferences for redistribution and the effect of culture. In contrast, labour immigrants from Poland and Turkey are likely to be self-selected into migration and their individual traits made them ask for less redistribution from the very beginning. By comparing self-directed risk takers from Poland and Turkey with resettled individuals from Russia and Kazakhstan, we can gain insights into the magnitude of selection bias caused by individual traits.

## Measurement

### Preferences for redistribution

Redistribution preferences were measured in two waves of the German Socio-Economic Panel (G-SOEP) in 1997 and 2002, with no subsequent measurements. These data are well-suited to the research purpose: in 1997 the sample included a substantial number of new migrants, which allows for testing integration hypothesis. Additionally, this time frame avoids the potential confounding effect of the Great Recession, which began in in 2008, followed by other global challenges affecting economic instability and sense of individual existential insecurity.

Respondents were asked about their preferences regarding the role of government in providing various benefits and social services: “At present, a multitude of social services are provided not only by the state but also by private free market enterprises, organizations, associations, or private citizens. What is your opinion on this? Who should be responsible for the following areas?” Their responses covered several dimensions of financial security: “financial security in case of unemployment”, “financial security in case of illness”, “financial security of families”, “financial security for old age” and “financial security for persons needing care. Responses were recorded on a 5-point Likert-scale where the score of 1 indicated “only the state”, 2 “mostly the state”, 3 “state and private forces”, 4 “mostly private forces” and 5 “only private forces”. For ease of interpretation, the scale was reversed for the analysis: variables take a value of 1 for “only private forces” and of 5 for “only the state”.

### Explanatory variables

The primary explanatory variable in this research is time. The aim is to estimate how preferences for redistribution change over time for individuals who were born and initially lived in one culture, and then moved to another. To account for differences arising from self-interest, a set of individual characteristics associated with welfare, as well as the social and income situation was controlled. The baseline controls include age, gender, marital status, labour force status, education, occupation of the respondent, number of children, number of adults in the household, annual household income measured in euro, income per capita, unemployment rates in German states, gross and net transfers per capita that each state received from other states and the federal government in 1997 and 2002.

### Models

The study estimates the effect of time spent in the host country in two steps: between-subject and within-subject research designs.

1. The first step estimates the difference in preferences between West Germans, East Germans and the four migrant groups. It examines how time spent in a new culture influences changes in the preferences for redistribution for each group, treating West Germans as a reference. Specifically, the first model tests how immigrants’ preferences for redistribution at the group level changed between 1997 and 2002 compared to West Germans


$$\begin{array}{c} {RP}_{it}=\beta_{1\;}\left({East}_{i}\;\right)+\beta_{2\;}\left({Poles}_{i}\;\right)+\beta_{3\;}\left({Turks}_{i}\;\right)+\;\beta_{4\;}\left({Russians}_{i}\;\right)+\beta_{5\;}\left({Kazakhs}_{i}\;\right)\\ +\beta_{6\;}\left({Year\;2002}_{it}\;\right)+\beta_{7\;}\left({East}_{i}\;*{Year\;2002}_{it}\right)+\;\beta_{8\;}\left({Poles}_{i}\;*{Year\;2002}_{it}\right)\\ +\;\beta_{9\;}\left({Turks}_{i}\;*{Year\;2002}_{it}\right)+\;\beta_{10\;}\left({Russians}_{i}\;*{Year\;2002}_{it}\right)+\;\beta_{11\;}\left({Kazakhs}_{i}\;*{Year\;2002}_{it}\right)+\;\beta_{12}X_{it}+\;\varepsilon_{it} \end{array}$$ (1)

Where:

${RP}_{it}$: redistribution preferences of an individual i at time t,

${East}_{i}$: dummy variable indicating whether an individual I is from East Germany (1 = East German, 0 = other)

${Poles}_{i}$, ${Turks}_{i}$, ${Russians}_{i}$, ${Kazakhs}_{i}$: dummy variables for migrant groups (1 = member of a respective group, 0 = other)

${Year\;2002}_{it}$: dummy variable indicating the effect of time (0= 1997 year, 1=2002)

$X_{it}$ a vector of individual characteristics measured at both 1997 and 2002

$\varepsilon_{it}$ error term

The linear regression approach does not account for the ordered, categorical nature of the dependent variable, which could potentially overlook the nuances in the relationships between categories. To check the robustness of the results, alongside the OLS model, a probit model was also employed using a binary version of the dependent variable. In this model, the preferences for redistribution were recoded to indicate a preference for state responsibility (1 = “mostly the state” or “only the state”; 0 = otherwise).

From a methodological standpoint, using a probit model could be seen as a more appropriate choice compared to OLS. However, adopting a probit model would necessitate recoding the dependent variable into a dummy variable, which would fundamentally alter the nature of the dependent variable and lead to a substantial reduction in variation in the phenomena under study.

If recoded into a binary format, the dependent variable would only differentiate between two broad categories: government responsibility (distinguishing between ‘mostly the state’ and ‘only the state’) and private forces (distinguishing between ‘only private,’ ‘mostly private,’ and ‘state and private’). This recoding would result in the loss of subtle distinctions, such as those between ‘state and private’ and ‘only private,’ or ‘mostly the state’ and ‘only the state.’ These are meaningful shifts that could provide important insights into the dynamics of immigrant integration. Small but significant changes in preferences, especially in areas like the balance between state and private sector responsibility, are critical to fully understanding the change in preferences showing integration process.

For this reason, it is essential to retain as much variation as possible in the dependent variable. By keeping the original ordered categories intact, OLS models allow for the preservation of this variation, enabling a more nuanced understanding of how immigrant integration evolves. Therefore, in this paper, the OLS models are considered the principal method, while probit models are employed as supplementary to test the robustness of the findings. This approach ensures that the analysis maintains its focus on the full spectrum of variation in the dependent variable, which is particularly important in studying complex processes like immigrant integration.

2. The second step implements a within-subjects research design. Current literature in econometrics suggests using fixed effects models to address the omitted variables bias [20, p. 247], identify causality, and estimate the effect of change in the independent variable [21,22]. Within subject estimation, compared to between subjects’ estimation, can resolve two key issues: selection into treatment and unobserved unit heterogeneity. Even if a subject self-selects into a survey these unobserved characteristics are eliminated by the fixed effects approach. This method controls for time-constant unobserved heterogeneity, including individual values [23].

Given that there are only two time points, either a first-difference or a fixed-effect model can be used to examine the within-subject dynamics of preferences for redistribution among West Germans and compare both East Germans and immigrants with them. Fixed effects models remove between-individual variation, isolating the effect of parameters that have been changed over time. In this context, averages for dependent variables and time varying parameters are demeaned. Correspondently, this model estimates how change in one parameter influences changes in the dependent variable.

Initial model tests the time effect (2) separately for West Germans, East Germans and four groups of migrants. The next model (3) adds time-varying controls such as income, number of family members, marital status and years of education.

At this step, the models take the following form:


$$\left(\text{RPit}-\overset{―}{\text{RP}}\text{i})\;=\;\beta (Year\;2002it-\overset{―}{{Year\;2002}_{i}})+(\varepsilon_{it}-{\overset{―}{\varepsilon }}_{i}\right)$$ (2)

$$\left(\text{RPit}-\overset{―}{\text{RP}}\text{i}\right)=\;\beta \left(Year\;2002it-\overset{―}{{Year\;2002}_{i}}\right)+\gamma (X_{it}-\overset{―}{X_{i}})+\left(\varepsilon_{it}-{\overset{―}{\varepsilon }}_{i}\right)$$ (3)

$X_{it}$ – is a vector of individual characteristics varying in time

While the fixed-effects linear regression model is used in this study to account for individual heterogeneity and control for unobserved time-invariant factors, there are limitations to this approach. The major limitation is the same as discussed previously, the ordinal nature of the dependent variable. Linear models, by design, assume numerical variables, and thus do not fully meet the methodological requirements for handling ordinal data. While one might consider substituting the fixed-effects linear regression with a fixed-effects logit model, this approach would lead to the loss of substantial variation in the dependent variable as on the previous step. Given the nature of our research, preserving this variation is essential. Additionally, the fixed-effects logit model presents its own challenges. It requires a likelihood-based estimation, which, when applied to a small-time dimension (t = 2), may suffer from incidental parameter bias and the loss of observations. In this study, the sample of migrants is sufficient to use linear fixed effects models, but using fixed-effects logit models reduces the number of observations by two-thirds, making the fixed-effects logit model less suitable.

For these reasons, the fixed-effects linear regression model is preferred in this analysis, as it better preserves variation in the dependent variable while addressing the issue of individual heterogeneity. However, the limitations associated with this model, especially in the context of ordinal data, should be considered when interpreting the results.

### Findings

Table 1 presents differences in preferences for redistribution between West Germans, East Germans, and migrant groups in 1997, as well as changes over time. To ensure comparability, the sample includes only individuals who did not migrate between East and West Germany.

**Table 1:** Comparisons of preferences for redistribution of migrants and West Germans over time.

	(1)	(2)	(3)	(4)	(5)	(6)	(7)	(8)	(9)	(10)
VARIABLES	When Unemp.	When Sick	For Fa- mily	In Old- Age	When Need Care	When Unemp.	When Sick	For Fa- mily	In Old- Age	When Need Care
	OLS					Probit				
East in East	0.288***	0.241***	0.259***	0.255***	0.223***	0.453***	0.393***	0.391***	0.405***	0.370***
	(0.020)	(0.020)	(0.021)	(0.021)	(0.019)	(0.034)	(0.032)	(0.032)	(0.032)	(0.032)
Turks (West)	0.040	0.221***	0.196***	0.188***	0.108**	−0.026	0.230***	0.263***	0.184***	0.086
	(0.043)	(0.042)	(0.045)	(0.044)	(0.042)	(0.068)	(0.066)	(0.066)	(0.066)	(0.066)
Poles (West)	0.024	0.159**	0.091	0.070	0.097	0.026	0.186*	0.014	0.018	0.110
	(0.072)	(0.062)	(0.069)	(0.074)	(0.072)	(0.110)	(0.106)	(0.109)	(0.106)	(0.105)
Russians (West)	0.107	0.271***	0.190**	0.173**	0.178**	0.360**	0.325**	0.216	0.216	0.282*
	(0.076)	(0.089)	(0.093)	(0.082)	(0.086)	(0.167)	(0.146)	(0.146)	(0.146)	(0.145)
Kazakhs (West)	0.240**	0.227**	0.175	0.020	0.106	0.173	0.161	−0.005	−0.258*	0.003
	(0.093)	(0.096)	(0.112)	(0.095)	(0.103)	(0.158)	(0.146)	(0.151)	(0.153)	(0.147)
Year 2002	0.011	0.090***	0.001	−0.017	0.042***	0.059**	0.155***	−0.020	−0.050*	0.098***
	(0.016)	(0.015)	(0.017)	(0.016)	(0.015)	(0.026)	(0.026)	(0.027)	(0.026)	(0.025)
East in East 2002	−0.064***	−0.062***	0.003	−0.068***	−0.091***	−0.138***	−0.106***	−0.026	−0.090**	−0.149***
	(0.024)	(0.024)	(0.026)	(0.025)	(0.024)	(0.043)	(0.040)	(0.040)	(0.040)	(0.040)
Turks (West) 2002	−0.029	−0.131***	−0.138**	−0.006	−0.093*	−0.079	−0.107	−0.215**	0.046	−0.089
	(0.051)	(0.051)	(0.054)	(0.053)	(0.052)	(0.080)	(0.082)	(0.084)	(0.083)	(0.077)
Poles (West) 2002	−0.066	−0.164**	−0.143	−0.119	−0.180**	−0.136	−0.170	0.086	−0.163	−0.262*
	(0.102)	(0.083)	(0.105)	(0.097)	(0.086)	(0.150)	(0.133)	(0.155)	(0.154)	(0.148)
Russians (West) 2002	−0.094	−0.093	0.004	0.243**	−0.073	−0.316	0.054	0.119	0.367**	−0.111
	(0.115)	(0.119)	(0.107)	(0.112)	(0.104)	(0.225)	(0.186)	(0.173)	(0.173)	(0.174)
Kazakhs (West) 2002	−0.104	−0.148	−0.192	0.063	−0.078	−0.017	−0.045	−0.062	0.361*	−0.124
	(0.136)	(0.134)	(0.162)	(0.128)	(0.137)	(0.220)	(0.224)	(0.239)	(0.214)	(0.208)
Age	−0.008	−0.002	−0.014	−0.012	−0.013	−0.018	−0.021	−0.010	−0.039**	−0.025
	(0.012)	(0.012)	(0.013)	(0.012)	(0.012)	(0.020)	(0.019)	(0.019)	(0.019)	(0.019)
Age squared	0.000	0.000	0.000	0.000	0.000	0.000	0.000	0.000	0.001**	0.000
	(0.000)	(0.000)	(0.000)	(0.000)	(0.000)	(0.000)	(0.000)	(0.000)	(0.000)	(0.000)
Age cubed	−0.000	−0.000	−0.000	−0.000	−0.000	−0.000	−0.000	−0.000	−0.000**	−0.000
	(0.000)	(0.000)	(0.000)	(0.000)	(0.000)	(0.000)	(0.000)	(0.000)	(0.000)	(0.000)
College	−0.104*	−0.130**	−0.119**	−0.186***	−0.109*	−0.229**	−0.259***	−0.203**	−0.305***	−0.142
	(0.056)	(0.055)	(0.057)	(0.060)	(0.057)	(0.100)	(0.090)	(0.090)	(0.090)	(0.091)
Vocational	−0.035	−0.050	−0.106**	−0.107*	−0.064	−0.116	−0.146*	−0.173**	−0.198**	−0.094
	(0.053)	(0.052)	(0.054)	(0.058)	(0.055)	(0.096)	(0.086)	(0.086)	(0.086)	(0.087)
Secondary school	−0.023	−0.007	−0.025	−0.061	−0.025	−0.118	−0.065	−0.048	−0.088	−0.047
	(0.054)	(0.053)	(0.055)	(0.059)	(0.056)	(0.097)	(0.087)	(0.088)	(0.087)	(0.088)
Intermediate tech.	−0.086	−0.030	−0.158**	−0.130*	−0.059	−0.223*	−0.187*	−0.203*	−0.319***	−0.070
	(0.066)	(0.066)	(0.071)	(0.069)	(0.068)	(0.116)	(0.109)	(0.111)	(0.107)	(0.111)
In school	−0.006	0.029	−0.005	−0.180**	−0.036	−0.089	−0.161	0.034	−0.240*	−0.071
	(0.084)	(0.083)	(0.098)	(0.092)	(0.086)	(0.151)	(0.144)	(0.143)	(0.140)	(0.142)
Male	−0.028*	−0.054***	−0.004	−0.019	0.011	−0.062**	−0.059**	0.013	0.017	0.039
	(0.015)	(0.015)	(0.016)	(0.016)	(0.014)	(0.025)	(0.024)	(0.024)	(0.024)	(0.024)
Number of children	0.007	0.020**	0.038***	0.015	0.018**	0.018	0.039***	0.056***	0.029**	0.031**
	(0.009)	(0.009)	(0.010)	(0.010)	(0.009)	(0.016)	(0.015)	(0.014)	(0.015)	(0.014)
Number of adults	−0.002	0.034***	0.019**	0.029***	0.005	0.007	0.053***	0.011	0.038***	0.014
	(0.008)	(0.007)	(0.008)	(0.008)	(0.007)	(0.013)	(0.013)	(0.013)	(0.012)	(0.012)
Married	0.053**	0.032	0.014	0.026	0.046**	0.104***	0.090**	0.033	0.078**	0.092**
	(0.024)	(0.024)	(0.026)	(0.024)	(0.023)	(0.040)	(0.039)	(0.039)	(0.039)	(0.038)
Divorced	0.040	−0.038	0.017	0.013	0.050	0.059	0.017	0.030	0.077	0.126**
	(0.038)	(0.037)	(0.038)	(0.038)	(0.036)	(0.061)	(0.058)	(0.058)	(0.059)	(0.056)
Married, but separated	−0.011	−0.015	0.025	0.078	0.085	0.096	−0.005	0.051	0.147	0.178*
	(0.049)	(0.051)	(0.058)	(0.056)	(0.053)	(0.093)	(0.089)	(0.090)	(0.093)	(0.092)
Widowed	0.008	0.032	0.004	−0.032	0.058	0.023	0.056	−0.012	0.010	0.113*
	(0.039)	(0.038)	(0.042)	(0.039)	(0.037)	(0.065)	(0.063)	(0.064)	(0.062)	(0.062)
Logged HH income	−0.106***	−0.182***	−0.120***	−0.187***	−0.102***	−0.169***	−0.282***	−0.136***	−0.245***	−0.148***
	(0.017)	(0.017)	(0.018)	(0.018)	(0.017)	(0.030)	(0.029)	(0.028)	(0.029)	(0.028)
Civil servant	−0.114***	−0.158***	0.039	−0.059	−0.080**	−0.191***	−0.237***	0.021	−0.040	−0.133**
	(0.040)	(0.039)	(0.044)	(0.040)	(0.035)	(0.063)	(0.066)	(0.066)	(0.066)	(0.061)
Self-employed	−0.233***	−0.267***	−0.259***	−0.286***	−0.227***	−0.329***	−0.405***	−0.297***	−0.415***	−0.335***
	(0.036)	(0.033)	(0.038)	(0.035)	(0.032)	(0.055)	(0.057)	(0.057)	(0.058)	(0.055)
White collar	−0.049**	−0.057***	−0.022	−0.070***	−0.088***	−0.039	−0.069*	0.005	−0.066*	−0.103***
	(0.022)	(0.021)	(0.023)	(0.023)	(0.021)	(0.037)	(0.035)	(0.036)	(0.035)	(0.035)
Currently in education	0.014	−0.104*	−0.028	0.022	−0.040	−0.044	−0.191**	−0.000	−0.038	−0.116
	(0.058)	(0.055)	(0.059)	(0.060)	(0.053)	(0.095)	(0.090)	(0.090)	(0.091)	(0.090)
Unemployed	0.041	0.002	0.077**	0.022	−0.026	0.064	−0.011	0.126***	0.017	−0.055
	(0.029)	(0.030)	(0.032)	(0.033)	(0.031)	(0.052)	(0.049)	(0.048)	(0.049)	(0.048)
Retired	−0.016	−0.062	0.069	−0.021	−0.052	−0.120*	−0.139**	0.088	−0.002	−0.059
	(0.040)	(0.040)	(0.044)	(0.042)	(0.039)	(0.066)	(0.065)	(0.065)	(0.064)	(0.064)
Maternity	0.037	−0.014	0.041	−0.062	−0.058	0.038	−0.070	0.067	−0.109	−0.038
	(0.049)	(0.050)	(0.051)	(0.050)	(0.049)	(0.085)	(0.083)	(0.080)	(0.083)	(0.081)
Nonworking	−0.013	−0.026	0.090***	0.006	0.003	−0.058	−0.047	0.117***	−0.005	0.014
	(0.026)	(0.026)	(0.028)	(0.028)	(0.026)	(0.045)	(0.043)	(0.043)	(0.043)	(0.043)
Training	0.021	0.055	−0.043	0.104*	0.013	−0.021	0.132	−0.047	0.129	0.027
	(0.057)	(0.054)	(0.060)	(0.057)	(0.057)	(0.098)	(0.094)	(0.096)	(0.093)	(0.093)
Other nonworking	0.010	−0.079**	−0.013	−0.036	−0.117***	0.023	−0.134**	0.018	0.003	−0.144**
	(0.037)	(0.035)	(0.039)	(0.038)	(0.035)	(0.063)	(0.059)	(0.060)	(0.059)	(0.060)
Constant	4.855***	4.852***	4.447***	5.156***	4.583***	2.107***	2.318***	0.867**	2.426***	1.508***
	(0.233)	(0.232)	(0.255)	(0.245)	(0.228)	(0.391)	(0.382)	(0.380)	(0.382)	(0.374)
Observations	15,562	15,567	15,568	15,589	15,587	15,562	15,567	15,568	15,589	15,587
R-squared	0.042	0.054	0.042	0.054	0.030	0.453***	0.393***	0.391***	0.405***	0.370***

*Note*: Estimates are from linear models (columns 1–5) and probit models (columns 6–10) with robust clustered errors.

DV is measured on a scale of 1–5 (“1” corresponds to “only private forces”, “5” to “only the state”) in OLS, on scale 0/1 in probit podels (“1” corresponds to “the state”).

Robust standard errors in parentheses.

*** p < 0.01, ** p < 0.05, * p < 0.1.

**Fig 1:**
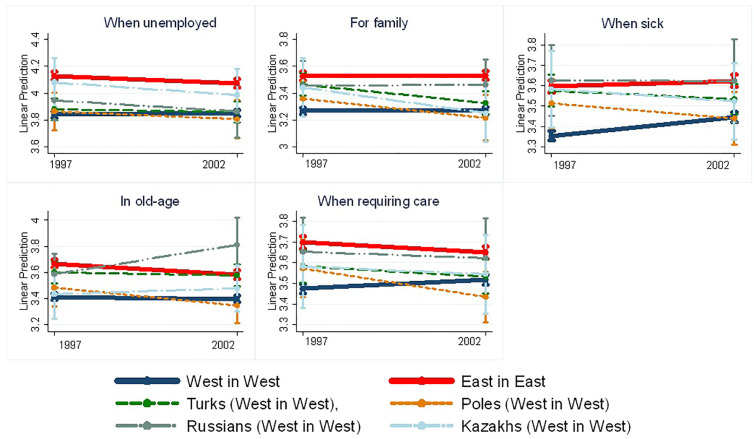
Preferences for redistribution of Germans and migrants in 1997 and 2002, linear predictions with 95% c.i., baseline controls.

In 1997, most migrant groups, particularly Turks, Kazakhs, Russians, and in some cases Poles, expressed a stronger preference for redistribution than West Germans. This pattern may reflect the influence of culture of their countries of origin, as suggested in previous research [2, p. 159], even after controlling for individual economic conditions. However, Polish migrants differed: their preferences in 1997 closely resembled those of West Germans in most domains. This similarity could result from self-selection into migration, where individuals with more liberal economic views are more likely to migrate and to choose a country like Germany [3]. Fig 1 shows marginal predictions and traces the evolution of preferences over time. The results reveal distinct integration trajectories across migrant groups and policy domains. While some groups display signs of convergence with West German preferences, the pace and extent of change vary considerably.

**Turkish migrants** initially in 1997 showed high support for redistribution in healthcare, family support, care and old-age domain. By 2002, their demand for redistribution in favour of sick, family and needing care declined (healthcare: −0.131; family support: −0.138; care: −0.091), indicating convergence with preferences of West Germans. However, their preference for redistribution in favour of old-aged remained elevated. Notably, Turkish migrants, similar to Polish migrants, were generally less supportive of redistribution toward the unemployed from the outset. These results suggest partial self-selection into migration among Turkish migrants, reflecting initially lower support for unemployment benefits. Over time, they appear to have assimilated in domains such as healthcare, family, and care support but maintained a distinct preference for redistribution in favour of the elderly.

**Polish migrants** appear to have integrated even more quickly. Their preferences were already close to West Germans in 1997 and became even more similar by 2002. Notably, Polish migrants reduced their support for needing care (–0.18), healthcare (−0,164), family (−0.143) and old-aged (−0,12), that indicate direct convergence. Their views on support unemployed was indistinguishable from West Germans from the very beginning like in case of Turkish migrants and remained like this until 2002. These findings confirm the idea that their alignment with West German preferences stems from both self-selection into migration and assimilation.

**Immigrants from Russia and Kazakhstan** followed slower and more heterogeneous adaptation trajectories. Initially, both groups showed stronger support for redistribution across nearly all social policy domains in 1997. However, Kazakh migrants exhibited more convergence than Russian migrants, suggesting differences in integration paths despite similar cultural and migration backgrounds. Interestingly, Kazakh migrants’ preferences regarding support for the elderly were already aligned with those of West Germans in 1997, possibly due to limited pension support in Kazakhstan during the 1990s, which may have shaped their lower expectations in this domain. By 2002, migrants from Kazakhstan had reduced their support in favour of the unemployed (−0.104), the sick (−0.148), families (−0.192), and slightly those in need of care (−0.08). However, as we can see in Fig 1, assimilation did not occur in all domains, but only in the support of family and those in need of care. We can notice some tendency towards integration in the domain of support for the unemployed and the sick. In contrast, support for the elderly increased, diverging further from West German preferences.

Migrants from Russia were more similar to East Germans in their initial preferences for redistribution and exhibited weaker assimilation to West German preferences. In 1997, their support for redistribution was consistently higher than that of West Germans across all domains. By 2002, these preferences had declined slightly, except in the domain of old-age support, where preferences even increased. Migrants from Russia thus show limited integration in attitudes toward unemployment, healthcare, and care, and continued divergence in preferences for support of the elderly and families.

To assess the robustness of these findings, **probit models** were employed alongside ordinary least squares (OLS) regressions. Initially, the dependent variable was measured on a 5-point Likert scale to capture preferences for redistribution across various social support domains. However, OLS assumes continuous dependent variable measured on an interval scale. To address this limitation, the ordinal dependent variables were recoded into binary outcomes: the categories “mostly the state” and “only the state” were combined into 1, reflecting stronger preferences for state support, while other categories were coded as 0, indicating a preference for private provision. This transformation facilitated the use of probit models and allowed for robustness checks.

The probit model in this study provided a more appropriate approach for analyzing categorical preferences in social policy domains. Comparing the OLS and probit models revealed consistent patterns, such as stronger demand for social support among East Germans compared to West Germans, recalling previous findings by Alesina and Fuchs-Schündeln [1]. This pattern was consistent also for migrants but in a more moderate way. Generally, probit models present the same direction and significance of the effects described above but make them slightly more explicit. These results highlight the value of using both OLS and probit models to capture the multifaceted influence of culture and time on preferences for redistribution. To conclude, the probit model confirms many of the OLS results, making our understanding of integration and assimilation more clear and reliable.

### Fixed effects models

Fixed-effects models with a within-subject design are employed to examine how preferences for redistribution evolve over time, from 1997 to 2002. This approach controls for time-constant parameters, such as unobserved individual characteristics, that remain stable over time, thereby isolating the effect of temporal changes on preferences. By focusing on within-individual comparisons, rather than between-individual differences, the method accounts for inherent, stable factors (e.g., political orientation, family background, basic human values) that might otherwise confound the results.

The results of the between-subjects test described above showed that, at the first survey, the subsample of migrants from Turkey, Russia and Kazakhstan showed higher demand for redistribution than those of West Germans in almost all domains. Except that migrants from Kazakhstan had similar demand for redistribution in favour of the elderly as West Germans, and migrants from Turkey in favour of the unemployed. The subsample of Poles represents a special case that can be described as self-selection into migration or country of migration. Poles initially had preferences similar to those of West Germans in almost all areas of social policy considered, with the exception of health care.

Table 2 presents the results of the within-subject analysis. The findings reveal that, over time, the preferences of migrants for redistribution tend to converge with those of West Germans, although the rate of convergence varies across different migrant groups and policy domains. The analysis below highlights these trends across migrant groups, focusing on their integration or assimilation with reference to their preferences.

**Table 2:** Effect of time on redistribution preferences for different subsamples of immigrants.

		Time effect, fixed-effects models			Time effect with controls for time varying parameters, fixed-effects models		
	Sample:	effect	St.err.	N (id year)	Effect	St.err.	N (id year)
When Unemployed	West	0.006	(0.016)	8815	0.014	(0.018)	8597
	East	−0.052***	(0.019)	5424	−0.019	(0.023)	5360
	Turks	0.007	(0.049)	915	0.024	(0.056)	893
	Poles	−0.109	(0.106)	279	−0.036	(0.142)	277
	Russians	−0.106	(0.117)	147	−0.061	(0.140)	142
	Kazakhs	**−0.175**	(0.146)	140	−0.085	(0.182)	138
When Sick	West	0.085***	(0.015)	8820	0.111***	(0.017)	8602
	East	0.026	(0.019)	5426	0.031	(0.024)	5362
	Turks	**−0.066**	(0.049)	915	−0.048	(0.057)	893
	Poles	**−0.070**	(0.084)	278	−0.014	(0.111)	276
	Russians	−0.031	(0.118)	146	−0.114	(0.156)	141
	Kazakhs	**−0.095**	(0.144)	140	0.080	(0.197)	138
For Family	West	−0.004	(0.017)	8818	0.021	(0.020)	8600
	East	0.002	(0.020)	5428	0.025	(0.025)	5364
	Turks	−0.138***	(0.053)	918	−0.102	(0.062)	896
	Poles	**−0.173**	(0.107)	275	−0.021	(0.144)	273
	Russians	−0.030	(0.108)	147	−0.168	(0.132)	142
	Kazakhs	**−0.238**	(0.175)	140	−0.331	(0.224)	138
In Old-Age	West	−0.023	(0.016)	8828	0.001	(0.018)	8609
	East	−0.082***	(0.020)	5436	−0.072***	(0.025)	5372
	Turks	−0.054	(0.051)	918	0.026	(0.059)	896
	Poles	−0.163	(0.099)	279	−0.166	(0.137)	277
	Russians	***0.167***	(0.109)	147	−0.023	(0.132)	142
	Kazakhs	−0.048	(0.135)	140	−0.073	(0.190)	138
Requiring Care	West	0.034**	(0.015)	8823	0.049***	(0.017)	8605
	East	−0.041**	(0.019)	5440	−0.024	(0.024)	5376
	Turks	−0.087*	(0.051)	917	−0.064	(0.059)	895
	Poles	−0.132	(0.089)	279	−0.149	(0.120)	277
	Russians	−0.045	(0.102)	147	−0.184	(0.131)	142
	Kazakhs	−0.111	(0.142)	140	−0.148	(0.192)	138

*Note*: Estimates are from fixed effects models. Estimates in the right column are controlled for: income, HH size and marital status. Dependent variable is measured on a scale of 1–5 (“1” corresponds to “only private forces”, “5” to “only the state”). Robust standard errors in parentheses, *** p < 0.01, ** p < 0.05, * p < 0.1

**Turkish migrants** displayed distinct patterns in their preferences for redistribution, with convergence evident in some domains but persistent differences in others. In 1997, Turkish migrants generally expressed stronger preferences for redistribution compared to West Germans. A notable exception was unemployment support, where their preferences were similar to those of the native population. Over time, their preferences in this domain remained stable, underscoring a baseline alignment with the host culture. Healthcare, on the other hand, was prioritized more highly by Turkish migrants in 1997, and this preference changed only marginally over five years. Despite small reductions in demand, significant alignment with West Germans’ views is projected to take 17 years (or 23 if we consider social and demographic characteristics) (See Table 3 presenting predicted years of integration). Migrants from Turkey show gradual decline in their support for measures focused on family, in this domain they were expected to be assimilated in 7 (or 10) years. Support for the elderly emerged as a particularly distinct domain for Turks. Turkish migrants consistently advocated for greater redistribution to the elderly, a preference that remained largely unchanged over time. Especially when we consider socio-demographic controls, convergence in this area is unlikely, reflecting deeply held cultural values. In contrast, preferences of Turkish migrants for care programs demonstrated elasticity. Over time, Turkish migrants reduced their demand for redistribution in this domain, with convergence predicted within 6 (or 8 with controls) years. These trends underscore the mixed trajectory of adaptation of Turkish migrants in Germany, with assimilation in their support for redistribution in favour of unemployed, family and requiring care, integration in the domain of healthcare preferences, and separation in the domain of redistribution to the elderly.

**Table 3:** Expected years for the convergence of preferences of immigrants and East Germans with the preferences of native West Germans in 1997. Estimates are based on fixed effects models (no controls/ with controls for a change in income and household composition).

	When Unemp.	When Sick	For Family	In Old-Age	When Need Care
**East in East**	28/76	No/ no	No/ no	16/18	27/46
**Turks (West)**	No/ no (similar)	17/23	7/10	17/ no	6/8
**Poles (West)**	1/3	11/57	3/22	2/2	4/3
**Russians (West)**	5/9	44/12	32/6	No/ 38	20/5
**Kazakhs (West)**	7/14	12/ no	4/3	2/1	5/4

**Polish migrants** present a unique case, as their preferences were initially quite similar to those of West Germans in most domains. By 1997, Polish migrants’ preferences for redistribution were already closely aligned with those of West Germans. This suggests a degree of self-selection, with migrants aligning with the host culture even before relocation. For preferences for unemployment, family, elderly and requiring care, their demand became even lower by 2002, indicating swift and assimilation. The single preferences of Poles that were significantly higher compared to West Germans in 1997 were in the healthcare domain. But also in this domain Poles demonstrate integration, complete assimilation is expected in 11 years, if we consider the main socio-demographic differences the process will take longer (57 year) but result in the end in assimilation.

**Russian migrants** exhibited higher initial preferences for redistribution across all the domains, with slower rates of change over time. In unemployment and care support, we can notice a trend to integration of preferences of Russian immigrants over the time. Russian migrants initially expressed significantly higher demand than West Germans and reduced it over five years: predictions claim that Russian immigrants will be assimilated in terms of their preferences for redistribution in favour of unemployed in 5 (or 9) years and in 5 years in favour of needing care when the key socio-demographic characteristics are controlled. Preferences for redistribution in other domains turned out to be notably stable. Convergence of preferences of Russian migrants and West Germans is predicted over a long horizon, however adjustments for socio-demographic factors sometimes shorten this timeline. Models predict assimilation of the preferences of Russian migrants in the healthcare domain in 44 years and family support domain in 32 years. Support for the elderly presented a unique pattern. Russian migrants’ demand for redistribution in this area increased over the study period, diverging further from West Germans, that indicates a clear case of separation. However, when accounting for socio-economic factors, preferences show potential for gradual alignment in 38 years.

**Kazakh migrants** demonstrated notable initial divergence from preferences of West Germans in preferences in the domains of unemployment, healthcare, family support, and care. By 2002, they had reduced their support of unemployed, with projections suggesting assimilation within approximately seven years (or fourteen years when accounting for socio-demographic differences). Similarly, their preferences in family support and care are expected to align with those of West Germans relatively quickly, with convergence projected in four (three) years for family support and five (four) years for care. In contrast, their support for redistribution in favour of the elderly showed initial alignment with West German preferences and little evidence of change over time. In contrast, preferences in healthcare domain changed minimally, indicating a prolonged divergence. Kazakh migrants initially expressed significantly higher support for redistribution in favour of the sick compared to West Germans and slightly reduced them over time. Projections suggest that assimilation in this domain happens in 12 years, but when socio-demographic controls are applied, alignment with West German preferences remains unlikely.

Although many estimates in the fixed-effects model are statistically insignificant due to the small sample sizes of immigrant subsamples, the overall findings support the assimilation hypothesis: over time, most migrants’ preferences are expected to align with those of West Germans, with a few exceptions. As labor migrants, Turkish and Polish immigrants initially exhibited levels of support for the unemployed similar to those of West Germans. Over time, Turkish migrants maintained this level of support, while Polish migrants further reduced it. However, significant divergences remain in other domains. For Turkish migrants, support for the elderly remains a particularly important issue, while healthcare preferences stand out for Polish migrants. These differences suggest that specific cultural or socio-economic factors continue to influence these groups’ preferences, even as they integrate in other areas.

Resettlers from Russia and Kazakhstan, although expected to follow similar integration trajectories, displayed some differences. Russian migrants exhibited slower assimilation than Kazakh migrants, particularly in domains such as support for the elderly, where they demonstrated a pattern of separation. Both groups emphasized the importance of healthcare, but Kazakh immigrants showed a trend of separation in this domain. This contrast highlights the complexity of assimilation processes and the potential influence of origin-country contexts, resettlement motivations, and host-country experiences on migrant preferences.

These findings reinforce the nuanced nature of integration, suggesting that while structural and economic factors play critical roles, cultural values also significantly influence the trajectory of preference alignment. Future research should explore the mechanisms behind these dynamics, providing deeper insights into the integration process.

## Conclusion

The central question of this paper was whether culture matters in shaping individual preferences for redistribution and whether dominant culture in the host country can transform individual the welfare attitudes of immigrants. Specifically, the paper explored whether immigrants can adopt preferences for redistribution typical for the population in a host country. To address this question, I examined immigrants in Germany, a country with distinct cultural and welfare environment. I focused on migrants from countries with significantly different preferences for redistribution and welfare arrangements. The G-SOEP made this study possible because it contains questions on the preferences for redistribution in favour of different social groups, panel data for Germany, including data for natives and migrants from different countries. I tested hypotheses derived from acculturation theory and self-selection approach.

Acculturation theory proposed different scenarios of adaptation of immigrants in a new culture from assimilation to marginalization. The self-selection approach predicted a difference between two groups of migrants: labour migrants and resettles. This study confirmed the main aspects of these theories. However, the data showed great complexity in the process of adopting a new culture by migrants.

Labor migrants, who freely choose their destination country, were expected to demonstrate more adaptive patterns of integration. This expectation was largely supported by the case of Polish migrants, whose initial support of the welfare provision for unemployed and the elderly was almost similar to that of West Germans from the very beginning (self-selection). Their preferences for family support and care also converged rapidly (assimilation). However, healthcare preferences displayed a slower integration process, with convergence driven by increasing demands for healthcare provision among West Germans.

In contrast, Turkish migrants, despite being labour migrants, did not show the same pattern of integration as the Poles. Their preferences in domains such as family support, care, and elderly support more closely resembled those of resettlers from Kazakhstan. Across these domains of social policy, both migrants from Turkey and Kazakhstan show a trend towards integration and assimilation at the end. But especially when it comes to health care, both migrants from Turkey and Kazakhstan were slow to adapt their preferences to those of West Germans. Results of both between-subject and within-subject estimates demonstrate it well. Interestingly, the convergence in healthcare preferences was largely due to rising demands among West Germans rather than assimilation by migrants.

The hypothesis that labour migrants are more adaptive to the host culture than resettles found further support in case of migrants from Russia, who didn’t belong to this group. The preferences for redistribution of Russian immigrants closely mirrored those of East Germans, who also experienced communism, and very slowly converged with preferences of West Germans.

The study concludes that no single scenario can fully explain the complex process of migrant adaptation. This process is a nuanced interplay between the cultures of origin and residence in shaping individual preferences. The self-selection hypothesis partially confirmed its relevance, highlighting distinct mechanisms of adaptation of migrants that complement the theory of acculturation. For example, Polish migrants demonstrated adaptation patterns driven by work incentives, while immigrants from Russia exhibited adaptation patterns shaped by historical, political and economic conditioning. Notably, even in the case of self-selection into migration and the country of migration, people may have fundamental preferences, such as those related to healthcare and pensions, on which they have a rather rigid position.

The data also showed a scenario that was not predicted by theory: the convergence of preferences typical for the dominant culture and the preferences of smaller groups of migrants may occur not due to the integration of the latter, but due to changes in the dominant culture itself. An unexpected finding was the bilateral convergence observed in preferences, where changes in the dominant culture contributed to alignment with migrant preferences. West Germans increased their demands for redistribution in favour of sick and needing care, driven potentially by political reforms under the Schröder government. This bilateral adaptation complicates the assessment of cultural effects, highlighting the dynamic interplay between host and migrant cultures in shaping welfare preferences.

From a methodological perspective, this study highlights the challenges of choosing an appropriate analytical approach for studying immigrant integration. While linear models were the primary method due to their ability to preserve the variation in the dependent variable, their use comes with certain limitations. Specifically, OLS and fixed effects models do not fully account for the ordered and categorical nature of preferences for redistribution measured on five-point Likert scale. To address this, probit models were employed as a robustness check for OLS estimates, utilizing a binary version of the dependent variable to capture preferences for redistribution. However, recoding the dependent variable into a binary format significantly reduces the variation in the data, potentially oversimplifying the nuanced shifts in preferences that are central to understanding the integration process. Fixed-effects logit models could be considered as a robustness check for fixed-effects linear models. However, this method poses practical challenges in the context of this study. Applying a fixed-effects logit model to the small-time dimension of the panel (t = 2) introduces incidental parameter bias and leads to a drastic reduction in sample size of migrants by approximately two-thirds. Given the already limited sample size of migrants such a loss of data makes the fixed-effects logit model unsuitable for data analysis.

The findings demonstrate that integration of immigrants is a complex, dynamic process, and the methodological choice to prioritize variation in the dependent variable ensures a more comprehensive understanding of these processes. The dual approach, using OLS for detailed analysis and probit models for robustness, provided a balanced framework for investigating immigrant integration in a nuanced and methodologically sound manner.

In summary, while the adoption of host culture preferences is a dominant trend, the pace and extent of integration are contingent on migration pathways, socio-economic contexts, and the centrality of specific welfare domains. Fundamental preferences for redistribution, rooted in historical and cultural experiences, demonstrate significant resilience, making their transformation a complex and gradual process. The persistence of distinct preferences in some dimensions aligns with Alba and Nee’s [24] notion that assimilation is not a monolithic process but varies based on contextual factors and the social distance between groups.

Future research on immigrant integration could greatly benefit from several methodological innovations and enhancements. First, the addition of more waves to longitudinal studies would allow for the examination of long-term integration trends and the dynamics of preference changes over extended periods. This would not only deepen our understanding of the complexities of adaptation processes but also provide an opportunity to test and validate previous findings and conclusions. Such an approach would strengthen the external validity of integration research by testing whether observed patterns hold over time and under changing conditions. Then, increasing the sample size of immigrant groups, particularly those underrepresented in current datasets, would improve the statistical power of analyses and allow for more granular comparisons. This would also enhance the reliability of findings by reducing the risk of biases associated with small sample sizes. In addition, modifying the scale of the dependent variable to approximate an interval scale could address the limitations associated with ordinal data. Alternatively, future studies could explore the use of advanced modelling techniques specifically designed for ordinal data, such as ordered logistic regression or hierarchical models, to achieve greater methodological precision while retaining the richness of the data. Also, expanding the scope of research to include diverse cultural and institutional contexts would allow for comparative studies across multiple host countries, providing insights into how different welfare systems, cultural norms, and migration policies shape integration patterns. Such comparisons could further test the generalizability of current findings, thereby contributing to the external validity of the research.
